# The Third dose of CoronVac vaccination induces broad and potent adaptive immune responses that recognize SARS-CoV-2 Delta and Omicron variants

**DOI:** 10.1080/22221751.2022.2081614

**Published:** 2022-06-02

**Authors:** Yuxin Chen, Lin Chen, Shengxia Yin, Yue Tao, Liguo Zhu, Xin Tong, Minxin Mao, Ming Li, Yawen Wan, Jun Ni, Xiaoyun Ji, Xianchi Dong, Jie Li, Rui Huang, Ya Shen, Han Shen, Changjun Bao, Chao Wu

**Affiliations:** a Department of Laboratory Medicine, Nanjing Drum Tower Hospital, Nanjing University Medical School, Nanjing, People’s Republic of China; b Institute of Viruses and Infectious Diseases, Nanjing University, Nanjing, People’s Republic of China; c Department of Laboratory Medicine, Nanjing Drum Tower Hospital Clinical College of Nanjing Medical University, Nanjing, People’s Republic of China; d Department of Infectious Diseases, Nanjing Drum Tower Hospital, Nanjing University Medical School, Nanjing, People’s Republic of China; e Jiangsu Provincial Center for Disease Control and Prevention, Nanjing, People’s Republic of China; f State Key Laboratory of Pharmaceutical Biotechnology, School of Life Sciences, Nanjing University, Nanjing, People’s Republic of China; g Engineering Research Center of Protein and Peptide Medicine, Ministry of Education, Nanjing, People’s Republic of China

**Keywords:** COVID-19 vaccine, booster, coronavac, neutralization, T cell responses

## Abstract

The waning humoral immunity and emerging contagious severe acute respiratory syndrome coronavirus 2 (SARS-CoV-2) variants resulted in the necessity of the booster vaccination of coronavirus disease 2019 (COVID-19). The inactivated vaccine, CoronaVac, is the most widely supplied COVID-19 vaccine globally. Whether the CoronaVac booster elicited adaptive responses that cross-recognize SARS-CoV-2 variants of concern (VoCs) among 77 healthy subjects receiving the third dose of CoronaVac were explored. After the boost, remarkable elevated spike-specific IgG and IgA responses, as well as boosted neutralization activities, were observed, despite 3.0-fold and 5.9-fold reduced neutralization activities against Delta and Omicron strains compared to that of the ancestral strain. Furthermore, the booster dose induced potent B cells and memory B cells that cross-bound receptor-binding domain (RBD) proteins derived from VoCs, while Delta and Omicron RBD-specific memory B cell recognitions were reduced by 2.7-fold and 4.2-fold compared to that of ancestral strain, respectively. Consistently, spike-specific circulating follicular helper T cells (cTfh) significantly increased and remained stable after the boost, with a predominant expansion towards cTfh17 subpopulations. Moreover, SARS-CoV-2-specific CD4^+^ and CD8^+^ T cells peaked and sustained after the booster. Notably, CD4^+^ and CD8^+^ T cell recognition of VoC spike was largely preserved compared to the ancestral strain. Individuals without generating Delta or Omicron neutralization activities had comparable levels of CD4^+^ and CD8^+^ T cells responses as those with detectable neutralizing activities. Our study demonstrated that the CoronaVac booster induced broad and potent adaptive immune responses that could be effective in controlling SARS-CoV-2 Delta and Omicron variants.

## Introduction

The high degree of waning humoral immunity and emerging contagious SARS-CoV-2 variants resulted in the occurrence of breakthrough infection [1] and the necessity of the booster vaccination of coronavirus disease 2019 (COVID-19). A recent real-world study in Israel suggested that the immunity against SARS-CoV-2 across all age groups was decreased a few months later after the second dose of immunization [2]. Meanwhile, SARS-CoV-2 Delta and Omicron variants have rapidly achieved widespread community transmission, accounting for most infections globally [3, 4]. Particularly, Omicron harbors 30–40 mutations in spike protein, including some substitutions which were previously confirmed to increase viral transmission and resist neutralizing antibodies [5]. Due to the reduced efficacy of the initial rollout of mass vaccination campaigns, the necessity of a third booster dose is constantly a concern [6].

There is still an ongoing debate about whether there is a need for booster vaccines owning to a lack of experiment evidence [7]. There is no consensus on the necessity of a third dose booster of COVID-19 vaccines in the whole population [8]. Nevertheless, WHO has recommended a third dose of inactive virus vaccine or a heterologous booster for people aged over 60 years who already have received the two-dose scheme [9] because of the pronounced decrease of neutralizing antibody (NAb) titres [10,11] and reduced effectiveness in the older population [12]. A booster dose of the BNT162b2 vaccine could significantly lower the rates of confirmed COVID-19 and severe illness across age groups [13]. The third-dose vaccine developed by Pfizer-BioNTech [14], Oxford-AstraZeneca [15] and Sinovac [10] induced surging levels of infection-blocking “neutralizing” antibodies. Among them, CoronaVac, a whole-virion inactivated vaccine produced by Sinovac, is the most widely offered COVID-19 vaccine globally [16]. Currently, a third booster dose of CoronaVac has been implemented among high-risk populations in China and other countries. However, there is little research on the protective immune responses elicited by the CoronaVac boosters against variants of concern since the vaccine is being applied in countries deficient in research capacity and resources [10]. Meanwhile, the impact of the variant-associated mutations has been established for most variants regarding antibody reactivity [17–19], while much is less available for vaccine-induced T cell and B cell responses. Additionally, whether a third dose could effectively boost the waned humoral and cellular immunity remains unclear. Therefore, it is urgent to evaluate the cross-reactivity of SARS-CoV-2 humoral and cellular responses against the emerging variants elicited by CoronaVac booster, so as to provide essential data for the public health response to the COVID-19 pandemic.

We have reported the dynamic antibody, B cell and T cell responses following immunization of CoronaVac in a prospective cohort of 100 SARS-CoV-2 naïve healthcare professionals [20,21]. It was revealed that 2-dose immunization effectively elicited spike-specific B cells, as well as SARS-CoV-2-specific CD4 ^+ ^T cell and CD8^+^ T cell responses. After 9 months, the third dose of CoronaVac booster was administered to 77 out of 100 healthcare professionals. In this study, their circulating antibody response, serum neutralization capacity, cellular responses including B cells, circulating T follicular helper cells (cTfh), as well as CD4^+^ and CD8^+^ T cell responses were closely monitored. The results demonstrated a necessity of a booster dose of CoronaVac, which can induce broad and potent adaptive immune responses effective in controlling SARS-CoV-2 Delta and Omicron variants.

## Materials and methods

### Study cohort and sample collection

**Figure 1.**
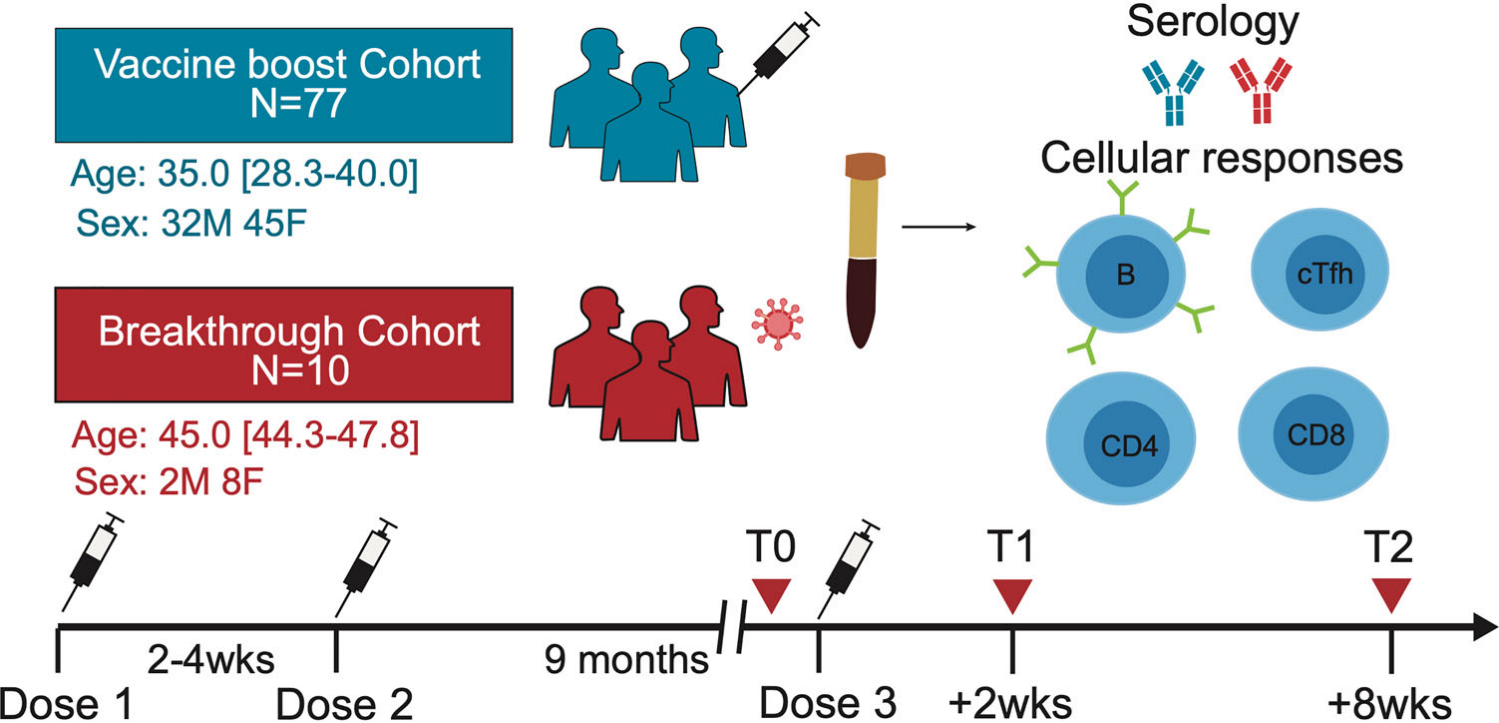
Study design and cohort summary.

Previously, we conducted a prospective, observational study (NCT04729374) in Nanjing Drum Tower Hospital, Jiangsu, China. All participants were tested negative for SARS-CoV-2 infection at screening and provided written informed consent. The clinical trial protocol was approved by the hospital ethics committee (2021-034-01). After 9 months after two-dose, the third dose of CoronaVac was administered to 77 healthcare professionals during the period from 8 Nov to 14 Nov 2021. Serum samples for detailed immunological assessments were taken at three different time points, including before the third dose (T0), day 14 post the third dose (T1), and day 56 post the third dose (T2) (Figure 1). The antibody titre and serum neutralization activity from this cohort were also compared to that of a breakthrough infection cohort, consisting of 10 subjects with Delta breakthrough infection after the two-dose vaccine from CoronaVac. Sera from the breakthrough cohort were obtained between day 13 and 18 post disease onset.

### Peripheral blood sample processing

Blood samples were collected via phlebotomy in acid citrate dextrose serum separator tubes, or ethylenediaminetetraacetic acid (EDTA) anticoagulated tubes. Peripheral blood mononuclear cells (PBMCs) were isolated from blood collected in EDTA tubes by lymphocyte separation medium density gradients (Stemcell Technologies, Vancouver, Canada) and resuspended in PRMI 1640 medium supplemented with 10% fetal calf serum (FCS), 1% penicillin/streptomycin and 1.5% HEPES buffer (Thermo Fisher Scientific, MA, USA) for stimulation assays or stored at – 135°C until used.

### Proteins and peptides

Pools of 15-mer peptides overlapping by 11 amino acid and together spanning the entire sequence of SARS-CoV-2 spike glycoprotein (S) from ancestral, Alpha (B.1.1.7). Delta (B.1.617.2) and Omicron (B.1.1.529) variants, wild-type virus open reading frame 3a, ORF7 and ORF8 (ORF3a/7/8), membrane protein (M), and envelope small membrane (E) were synthesized (Genscript, Jiangsu, China) and used for *ex vivo* stimulation of PBMCs.

The ectodomain of the ancestral SARS-CoV-2 spike (GenBank: MN908947.3) was expressed as previously described [22]. The prefusion Omicron (B.1.1.529/21 K) spike ectodomain (GenBank: OL672836.1, residues 1-1205) was cloned into vector pcDNA3.1 (Thermo Fisher Scientific, MA,USA) with proline substitutions at residues 983 and 984, a “GSAS” instead of “RRAR” at the furin cleavage site (residues 679-682), with a C-terminal T4 fibritin trimerization motif, an HRV-3C protease cleavage site, a Twin-Strep-tag, and an 8×His-tag according to Jason S. McLellan’s research [23]. The protein was purified from FreeStyle 293-F cells (Thermo Fisher Scientific, MA, USA) using affinity chromatography followed by size exclusion chromatography, detailed as described previously [22].

### Measurement of SARS-CoV-2 spike and RBD-specific IgG and IgA titre

Antigen-specific serological antibodies against SARS-CoV-2 were determined by enzyme-linked immunosorbent assay (ELISA) [20,21]. Briefly, 96-well plates were coated with 500 ng/mL of each recombinant viral antigen overnight. The plates were incubated with serum samples at a dilution of 1:200, followed by incubation with either anti-human IgG conjugated with HRP (ab6759, Abcam, Cambridge, England) or anti-human IgA conjugated with HRP (ab97215, Abcam, Cambridge, England). Subsequently, the plates were incubated with TMB substrate for 1 h and the reaction stopped with 1M H_2_SO_4_. Optical density (OD) value at 450 nm was measured. The cut-off value was determined as the average of OD values plus 2 standard deviations (SD) from 45 archived healthy individuals from the year of 2019 as the unexposed donors. Antibody endpoint titre was determined by the highest dilution of serum which gives an OD value higher than cut-off value of the healthy control group at the same dilution.

### Pseudovirus neutralization assay

Pseudovirus neutralization assay was performed as previously described to evaluate the serum neutralization capability that highly correlated with authentic neutralization assay [20,24]. Briefly, the lentivirus-based SARS-CoV-2 pseudoviruses were provided by Vazyme Biotech Co.,Ltd (Nanjing, China), which bear the spike protein derived from the D614G variant, the Delta variant (B.1.617.2), and the Omicron variant (B.1.1.529). SARS-CoV-2 pseudovirus was produced by co-transfection of a HIV-1 NL4-3 luciferase reporter vector that contains defective Nef, Env and Vpr (pNL4-3.luc.RE) and a pcDNA 3.1 expression plasmid (Invitrogen, Thermo Fisher Scientific, MA,USA) encoding respective spike protein in 293T cells. After 48 h, cell supernatants containing pseudoviruses were collected, filtered, and stored at −70˚C until use. The 50% tissue culture infectious dose (TCID_50_) of SARS-CoV-2 pseudovirus was measured by luciferase assay in relative light units (RLUs). To determine the neutralization activity of vaccinee serum, three-fold serial dilution starting from 1:30 were performed for heat-inactivated serum samples in duplicated before adding 1 × 10^3^ TCID_50_ pseudoviruses per well for 1h, together with the virus control and cell control wells. The mixture was added to 2 × 10^4^ HEK293T-ACE2 cells (Cat# DD1401-01, Vazyme, Nanjing, China) per well and incubated for 48 h in 5% CO_2_ environment at 37˚C. The luminescence was measured using Bio-lite Luciferase assay system (Cat# DD1201-01, Vazyme, Nanjing, China) and detected for RLUs using a Spark multimode microplate reader (Tecan, Männedorf, Switzerland). The titre of neutralization antibody (ID_50_) was defined as the reciprocal serum dilution at which the relative light units (RLUs) were reduced by 50% compared to the virus control wells after background RLUs in the control groups with cells only were subtracted. Data for pseudovirus neutralization titres for the D614G variant after 2-dose CoronaVac were reported previously [20].

### Antigen-Specific measurement of cellular analysis

Antigen-specific measurement of cellular analysis was performed as previously described [21]. For RBD-specific B cell analysis, PBMC samples were incubated with 100 ng fluorescence APC or PE-labeled RBD protein at 4°C for one hour to ensure maximal staining quality, followed by surface staining with antibodies at 4°C for 30 min. The following antibodies for phenotypic B cell surface markers were used, including anti-CD19-BV421 (Clone HIB19, 1:50), anti-CD27-BV655 (Clone MT-271, 1:50), anti-CD45-PE-cy7 (Clone HI30, 1:50), anti-CD3-Percp-cy5.5 (Clone OKT3, 1:50), anti-CD14-Percp-cy5.5 (Clone rmC5-3, 1:50), anti-CD16-Percp-cy5.5 (Clone B73.1,1:50), anti-CD56-Percp-cy5.5 (Clone B159, 1:50), anti-IgD-FITC (Clone IA6-2,1:50). Fixable viability Dye eFluor 780 staining was used to exclude dead cells. The above antibodies were purchased from BD Biosciences (San Diego, USA). The frequency of circulating RBD-specific B cells was expressed as the percentage of total B cells (CD19 ^+ ^CD20 ^+ ^CD3^-^CD14^-^CD16^-^CD56^-^LIVE/DEAD^-^lymphocytes). The frequency of antigen-specific RBD-specific memory B cells were expressed as a percentage of total memory B cells (CD19 ^+ ^CD20 ^+ ^CD27 ^+ ^CD3^-^CD14^-^CD16^-^CD56^-^LIVE/DEAD^-^lymphocytes). Gating strategy for B cell analysis is shown in Supplementary figure 1.

To measure antigen-specific circulating CD4^+^ T cells, CD8^+^ T cells and cTfh cells, activation-induced marker (AIM) assay was performed. Activation-induced marker (AIM) assay is a recently developed as a cytokine-independent method, capable of detecting early responding antigen-specific CD4 ^+ ^T cells, CD8^+^ T cells, and cTfh cells [25–27]. 1 × 10^6^ fresh PBMCs were suspended in Roswell Park Memorial Institute (RPMI) medium and stimulated with peptides pools at a final concentration of 1 μg/mL overnight. Stimulation with an equimolar amount of dimethylsulfoxide (DMSO) was performed as a negative control, and PMA/Ionomycin as a positive control. Following stimulation, cells were stained in flow buffer (DPBS, Gibco, NY, USA) supplemented with 2% FCS for 20 min at 4°C for viability. For CD4 ^+ ^T cell and CD8^+^ T cell analysis, the following antibodies were included for phenotypic lymphocyte surface markers: anti-CD3-PerCP-cy5.5 (Clone OKT3, 1:25), anti-CD4-Qdot655 (Clone RPA-T4, 1:50), anti-CD8-BV421 (Clone SK1, 1:25), anti-OX40-FITC (Clone L106, 1:50), anti-4-1BB-PE (Clone C65-485, 1:50), anti-CD45-PE-cy7 (Clone HI30, 1:50), anti-CD69-APC (Clone FN50, 1:25). Fixable viability Dye eFluor 780 staining was used to exclude dead cells. Gating strategy for T cells is shown in Supplementary figure S2. AIM^+^CD4^+^ T cells were defined based on the dual expression of 4-1BB and OX40, and AIM^+^CD8^+^ T cells were identified based on the dual expression of 4-1BB and CD69. The fraction of CD4^+^ or CD8^+^ T cells responsive to 5 overlapping peptide pools covering the ancestral spike glycoprotein, nucleoprotein (N), membrane protein (M), envelope small membrane protein (E), ORF3a/7/8 were then added as a combined sum of SARS-CoV-2 specific CD4^+^ or CD8^+^ T cells. For Tfh cell analysis, the following antibodies were used for phenotypic lymphocyte surface markers: anti-CD3-PerCP-cy5.5 (Clone OKT3, 1:25), anti-CD4-Qdot655 (Clone RPA-T4, 1:50), anti-OX40-FITC (Clone L106, 1:50), anti-4-1BB-PE (Clone C65-485, 1:50), anti-CD45-BV421 (Clone HI30, 1:50), anti-CXCR5-CF594 (Clone RF8B2, 1:50), anti-CCR6-APC (Clone 11A9, 1:50), anti-CXCR3-PE-cy7 (Clone 1C6, 1:50). Fixable viability Dye eFluor 780 staining was used to exclude dead cells. Spike-specific cTfh cells were gated as OX40^+^4-1BB^+^CXCR5 ^+ ^CD4 ^+ ^T cells and were further divided into cTfh1(CXCR3 ^+ ^CCR6^-^), cTfh2(CXCR3^-^CCR6^-^), cTfh17(CXCR3^-^CCR6^+^) and cTfh1-17(CXCR3 ^+ ^CCR6^+^). Gating strategy for cTfh cells is shown in Supplementary figure S3. Antigen-specific CD4^+^ T cell, CD8^+^ T cell and cTfh cell responses determined by AIM assays were calculated as background (DMSO) subtracted data. The above antibodies were purchased from BD Biosciences (San Diego, USA). The lower limit of detection (LOD) for cellular analysis was calculated using the median two-fold standard deviation of all negative control samples from the unexposed donors. Staining samples were analyzed by a fluorescence-activated cell sorter (FACS) Aria™ III Cell Sorter instrument (BD Biosciences) using FlowJo software (version 10).

### Statistical analysis

Binding antibody titres or neutralization titres were expressed as geometric mean titres (GMTs). The mean (standard deviation (SD)) or median (interquartile range (IQR)) was used to present the continuous variables. Categorical variables were described as counts and percentages. Wilcoxon matched-pairs signed-rank was used for comparison between timepoints and between SARS-CoV-2 variants. Unpaired Wilcoxon test for comparison between groups. The correlation between 2 continuous variables was analyzed using the Spearman correlation analysis. *p* < .05 was considered statistically significant. *indicates *p* < .05, ** indicates *p* < .01, *** indicates *p*< .001, **** indicates *p*< .0001, and ns indicates no significant difference. SPSS software program version 22.0 (Chicago, IL, USA) was used for data analysis.

## Results

### Cohort design

A prospective observational study to follow vaccine-induced immune response previously characterized the longitudinal magnitude of antibody response, as well as CD4^+^ and CD8^+^ T cells in a prospective observational cohort that received two-dose of CoronaVac [20,21]. Seventy-seven healthy individuals from this original cohort received the third dose of CoronaVac 9 months after the priming two-dose vaccination (Figure 1 and Table 1). Specifically, sampling at pre-boost (T0), 2 weeks (T1) and 8 weeks (T2) after the third immunization of CoronaVac enabled the dynamic immune analysis of SARS-CoV-2-specific humoral responses, B cells, CD4^+^ T cells, CD8^+^ T cells and cTfh cells. Paired serum and PBMC samples were collected from all individuals, allowing detailed analyses of both serological and cellular immune responses to SARS-CoV-2 antigens derived from different variants. Furthermore, a control donor group was set for humoral responses, in which 10 subjects with Delta breakthrough infection were prior fully vaccinated with CoronaVac. In this way, the dynamics of re-activating pre-existing immunity elicited by SARS-CoV-2 inactivated vaccines were investigated.

**Table 1.** Baseline clinical characteristics of the CoronaVac booster cohort and the breakthrough infection cohort.

	CoronaVac booster group	Breakthrough infection group
	(*n* = 77)	(*n* = 10)
Sex		
Male	32 (41.6)	2 (20%)
Female	45 (58.4)	8 (80%)
Age (years)		
Median age (IQR)	35.0 (28.3, 40.0)	45.0 (44.3, 47.8)
Age group, years		
18–29	26 (33.8)	0 (0%)
30–39	30 (39.0)	0 (0%)
40–49	14 (18.1)	9 (90%)
50–59	7 (9.1)	1 (10%)
Sample types	Serum and PBMC	serum
Interval between 1st and 2nd dose of CoronaVac (days)		
Median (IQR)	21 (17.25, 22.75)	22 (17.0, 38.5)
Booster or infection since the 2nd dose of CoronaVac (months)		
Median (IQR)	8.43 (8.03, 8.52)	2.37 (1.33, 3.73)

### Boosted antibody responses to SARS-CoV-2 Ancestral, Delta and Omicron spike antigens

**Figure 2.**
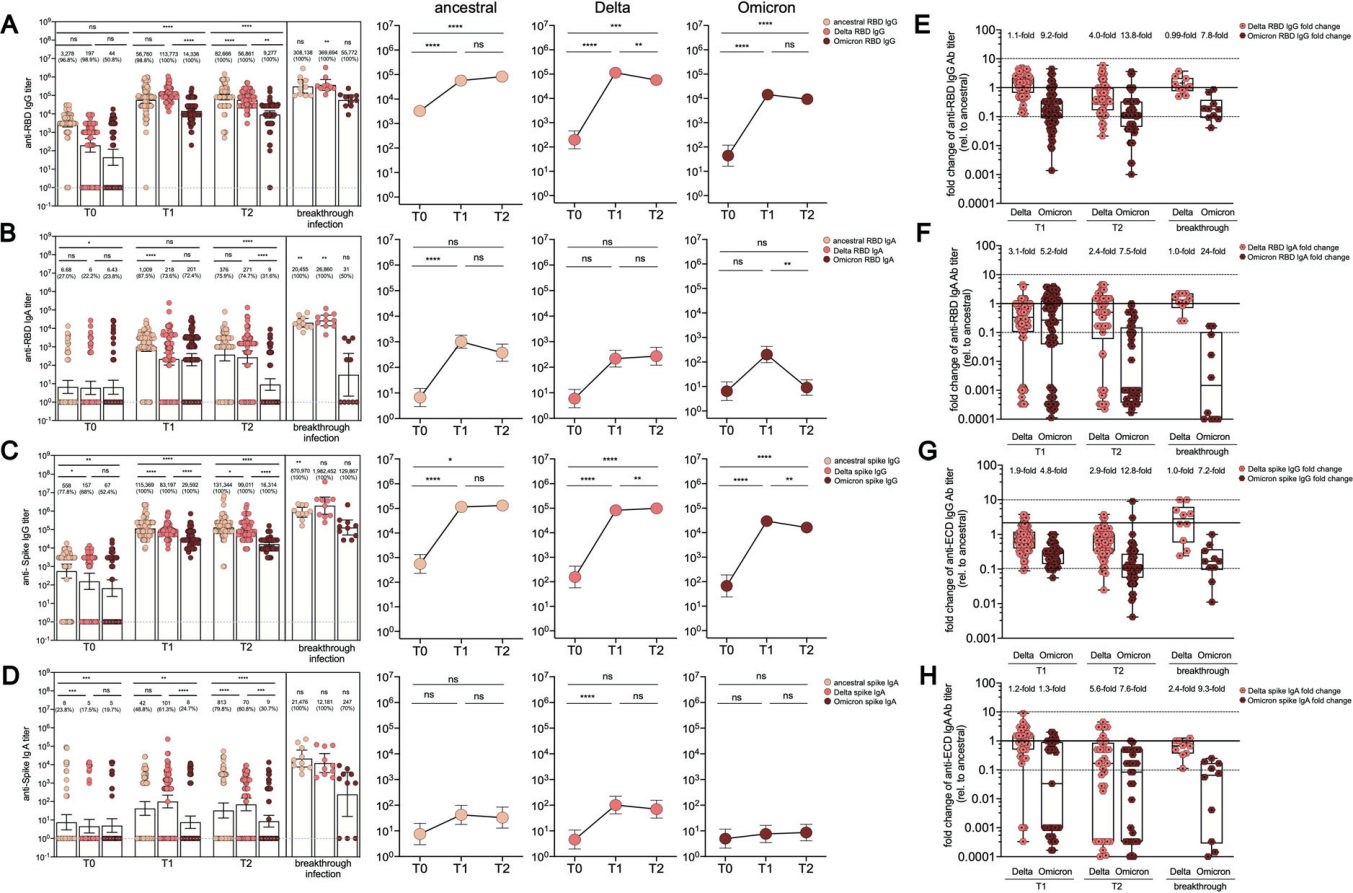
Dynamic anti-RBD or anti-spike antibody responses before and after the third CoronaVac booster. (A-D) Enzyme-linked immunosorbent assay measurement for anti-RBD IgG titre (A), anti-RBD IgA titre (B), anti-spike IgG titre (C) and anti-spike IgA titre (D) at three different time points, including before the booster (T0), 2 weeks after the booster (T1), and 8 weeks after the booster (T2) for vaccine booster group. Serum from a breakthrough cohort was also included for analysis as control, which were obtained between day 13–18 post disease onset. Dotted lines indicated the lower limit of detection (LOD) for the assay. Data points on the bar graph represent individual titre and the line indicates geometric mean titre (GMT). GMTs and the seropositive ratios were noted on the top of each bar. (E–H) Fold change in anti-RBD IgG titre (E), anti-RBD IgA titre (F), anti-spike IgG titre (G), anti-spike IgA titre (H) specific to Delta or Omicron compared to that of ancestral strain for booster at T1 and T2 timepoint as well as for breakthrough cohort. For comparing antibody responses specific to different SARS-CoV-2 variants and at timepoints, two-tailed *p* values were determined using matched-pairs signed rank test with the Holm-Šídák multiple comparison correction. Unpaired Wilcoxon test for comparison between vaccine booster at T1 timepoint and breakthrough infection subjects. * *p* < .05, ** *p* < .01, *** *p* < .001, **** *p* < .0001; ns, no significant difference.

First, the pre- and post-boost IgG serum titres against the ancestral (Wuhan-1), Delta (B.1.617.2) and Omicron (B.1.1.529) RBD and spike proteins were measured in our vaccine cohort (Figure 2). A booster dose of CoronaVac elicited a strong recall response in all individuals, with increased anti-RBD and anti-spike IgG and IgA titres compared with pre-boost titres. Baseline sera exhibited an anti-ancestral RBD-specific binding immunoglobulin G (IgG) geometric median titre (GMT) of 3,278 (95% CI, 1,953-5,504), while GMT specific to Delta RBD was 197 (84–460) and omicron RBD was 44 (16–120), respectively. After the booster dose, anti-ancestral RBD-specific binding IgG was increased to a GMT of 56,760 (38,284–84,151); the GMTs for anti-Delta RBD IgG and anti-Omicron RBD IgG were 113,773 (94,925,136,363) and 14,336 (11,025,18,641), respectively. The IgG titres stayed stable for 2 months after the booster and the IgG GMTs specific to ancestral RBD, Delta RBD and Omicron RBD were 82,666 (57,308, 119,244), 56,861 (45,630, 70,856) and 9277 (5903, 14,581), respectively (Figure 2(A)). Notably, anti-Delta RBD-specific IgG titres at T1 and T2 timepoint were 1.1-fold and 4.0-fold lower compared to that specific to ancestral RBD, respectively (Figure 2(E)). The booster recipients presented 9.2-fold and 13.8-fold decreased anti-Omicron RBD-specific IgG titre compared to those of anti-ancestral RBD protein after 2 and 8 weeks, respectively. Meanwhile, a low level of anti-RBD IgA responses was detected. The anti-ancestral RBD IgA at baseline possessed a GMT of 6.7 (3.0–15.0), elevated to 1009 (562.8–1808) after the booster, and then dropped to 376 (173.3–814.1) 2 month after the booster (Figure 2(B,F)). Consistently, spike-specific IgG and IgA also followed a similar trend as RBD-specific IgG and IgA responses (Figure 2(C,D,G,F)). The breakthrough cohort, as a crucial control, demonstrated a comparable level of IgG responses specific to ancestral RBD (*p* = .18) and omicron RBD (*p* = .07) but significantly higher level of IgG responses specific to Delta RBD (6.48-fold, *p* = .02) compared to those of 3-dose recipients at T1 timepoint, respectively. Similarly, anti-Omicron RBD IgG titre was 7.8-fold lower than that of anti-ancestral RBD in the breakthrough cohort (Figure 2(E)). Besides, the breakthrough cohort exhibited a significantly higher level of IgA titre specific to ancestral RBD protein and Delta RBD protein but a comparable level of Omicron RBD-specific IgA responses, compared to the booster vaccine cohort at the T1 timepoint. Our data suggested that the booster dose can not only increase the magnitude of IgG and IgA responses but also broaden the antibody responses specific to spike protein derived from emerging viral variants.

### Improved potent and broad neutralization against emerging SARS-CoV-2 variants

**Figure 3.**
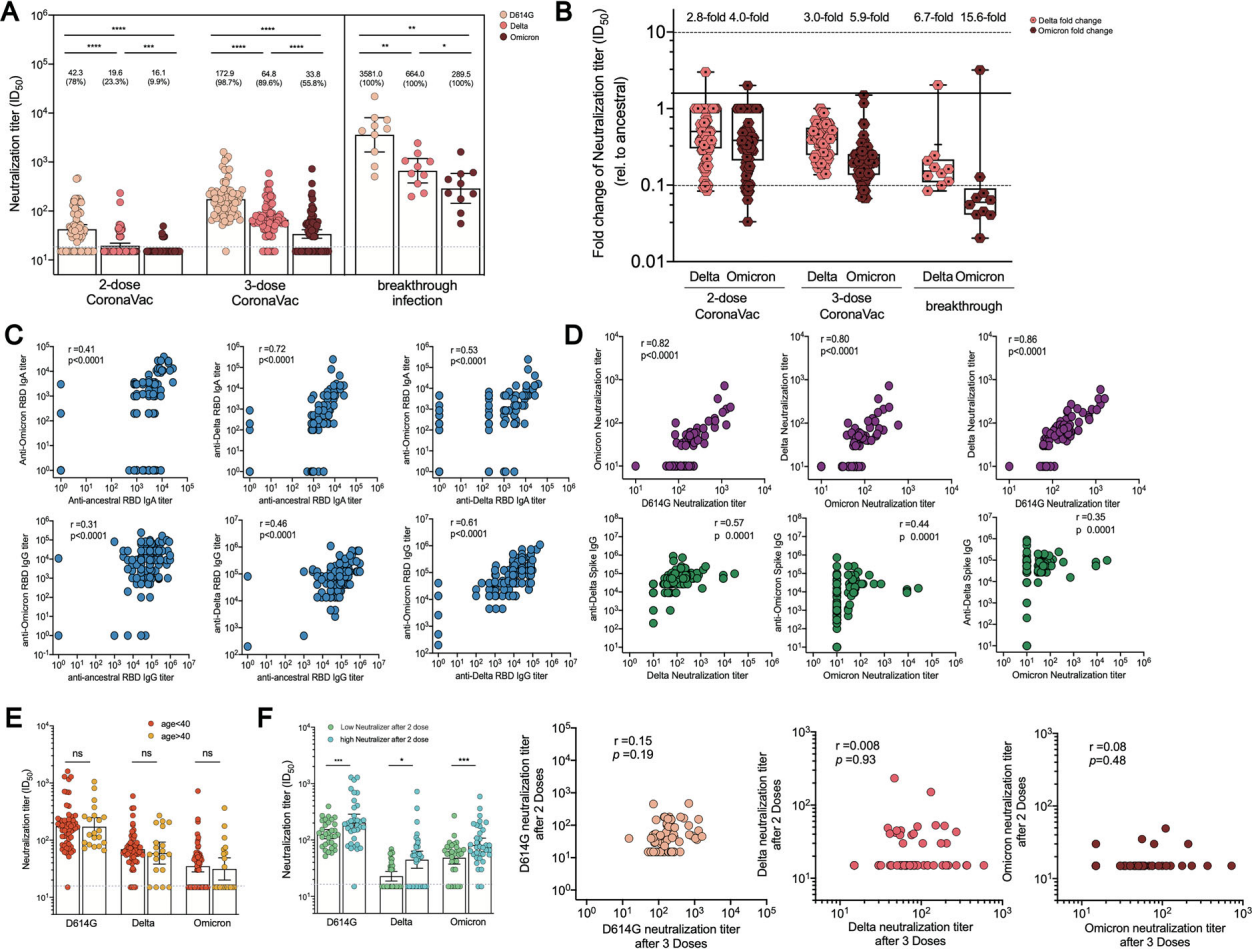
Serum neutralization activities in CoronaVac booster recipients and breakthrough infection cases. (A) Serum titres that achieved 50% peudovirus neutralization (ID_50_) in 77 CoronaVac booster recipients and 10 Delta breakthrough infection cases with prior 2-dose of CoronaVac. The vaccine sera were collected on day 14 post 2-dose and 3-dose of CoronaVac, respectively, while the breakthrough sera were obtained between day 13–18 post disease onset. The horizontal dotted lines indicate half the value of the lower limit of detection. Data points showed on the bar graph represent individual titre and the line indicates geometric mean titre (GMT). For pairwise comparison of serum samples collected after 2 and 3 doses, two-tailed *p* values were determined using matched-pairs signed rank test with the Holm-Šídák multiple comparison correction. Unpaired Wilcoxon test was used for comparison between groups. (B) Fold change in neutralization titre (ID_50_) for Delta and Omicron strain relative to that for D614G strain at different timepoint. The mean change fold was on the top of bar. (C) Correlation analysis of anti-RBD IgA or IgG responses specific to ancestral, Delta, and Omicron. (D) Correlation analysis of neutralization titre (ID_50_) against D614, Delta, and Omicron strain, correlation analysis of ID_50_ against Delta strain and anti-Delta spike IgG, and correlation analysis of ID_50_ against Omicron strain and anti-Omicron spike IgG or anti-Delta spike IgG responses. (E) Neutralization titres against D614G, Delta and Omicron strain among booster recipients under the age of 40 versus over the age of 40. (F) Neutralization titres against D614G, Delta and Omicron strains among low neutralizers after 2-dose CoronaVac versus high neutralizers after 2-dose CoronaVac. Correlation analysis for neutralization titres after 2 doses versus neutralization titres after 3 doses for D614G, Delta and Omicron strain. Correlation analysis was performed using nonparametric Spearman rank correlation. * *p* < .05, ** *p* < .01, *** *p* < .001, **** *p* < .0001; ns, no significant difference.

Our previous study reported that serum from 2-dose CoronaVac recipients in our cohort could effectively neutralize D614G and Alpha variants [20]. In this study, the neutralization titres against D614G, Delta, and Omicron variants were analyzed for serum collected at 2 weeks post 2-dose and 3-dose CoronaVac immunization to determine whether a third CoronaVac dose could increase the potency and breadth of serum neutralization activities (Figure 3(A)). Most (98.7%, 76/77) 3-dose recipients can neutralize against D614G with a GMT of 172.9 (141.8–210.9) compared to a GMT of 42.3 (34.1–52.5) after 2 doses. Meanwhile, the breakthrough infection cohort presented a surging neutralizing GMT of 3581.0 (1601.0–8012.0). Additionally, 89.6% (69/77) of booster sera neutralized against Delta strain (GMT 64.8, 53.4–78.5) with a 5.0-fold increase compared to serum samples from 2-dose vaccinees. However, the breakthrough infection resulted in a GMT of 664 (373.7–1181.0) against Delta strain. Booster recipients and breakthrough cohort demonstrated 3.0-fold and 6.7-fold less neutralization susceptible than D614G, respectively (Figure 3(B)). Meanwhile, 43 (55.8%) of subjects revealed neutralizing activities against Omicron strain after the boosting dose, with a 3.6-fold increased GMT from 16.1 (15.3–16.9) to 33.8 (27.7–41.5). All subjects with breakthrough infection possessed a neutralization capability with a GMT of 289.5 (143.5–584.1) for Omicron. Booster recipients and breakthrough cohort had 5.9-fold and 15.6-fold lower neutralization potency against the Omicron strain, respectively, compared to the ancestral strain. Our data suggested that a booster vaccine not only strongly enhances overall neutralizing potency against SARS-CoV-2 but also strengthens the broad recognition for D614G strain, Delta and Omicron strains.

Strong correlations were observed between the magnitudes of IgG or IgA responses specific to ancestral RBD and that of antibody responses specific to VoC RBD (Figure 3(C)). Consistently, serum neutralization titres for D614G, Delta, and Omicron strains were highly correlated with each other, respectively (Figure 3(D)). Delta neutralization titre was strongly correlated with anti-Delta spike IgG responses (*r *= 0.57, *p* < .0001). Omicron neutralization titre was moderately related to anti-Omicron spike IgG (*r* = 0.44, *p* < .001) and anti-Delta spike IgG (*r* = 0.35, *p* < .0001). Besides, the possible factors that might affect the neutralization activities were explored due to heterogeneous neutralization potency observed in our cohort. Our work [21] and other studies [28] have demonstrated age-dependent neutralization activities for SARS-Cov-2 ancestral and emerging VoCs among CoronaVac or mRNA recipients. Nevertheless, 3-dose recipients under the age of 40 and over the age of 40 generated comparable magnitudes of neutralization activities (Figure 3(E)). Furthermore, whether pre-existing neutralization activities might affect the boosted humoral immunity was assessed. The serum neutralization activities on week 2 after 2 doses of CoronaVac in this cohort were previously reported [20]. Our cohort was further divided into two groups: individuals with prior low neutralization activities (serum ID_50 _< 50) and individuals with prior high neutralization activities (serum ID_50_ ≥ 50). Our data implied that previous low neutralizers still had reduced serum neutralization activities compared to that of high neutralizers, even after the CoronaVac booster. Nonetheless, there was no correlation between neutralization titre after 2 doses and neutralization titre after 3 doses (Figure 3(F)).

### B cell responses to SARS-CoV-2 ancestral, Delta and Omicron RBD protein

**Figure 4.**
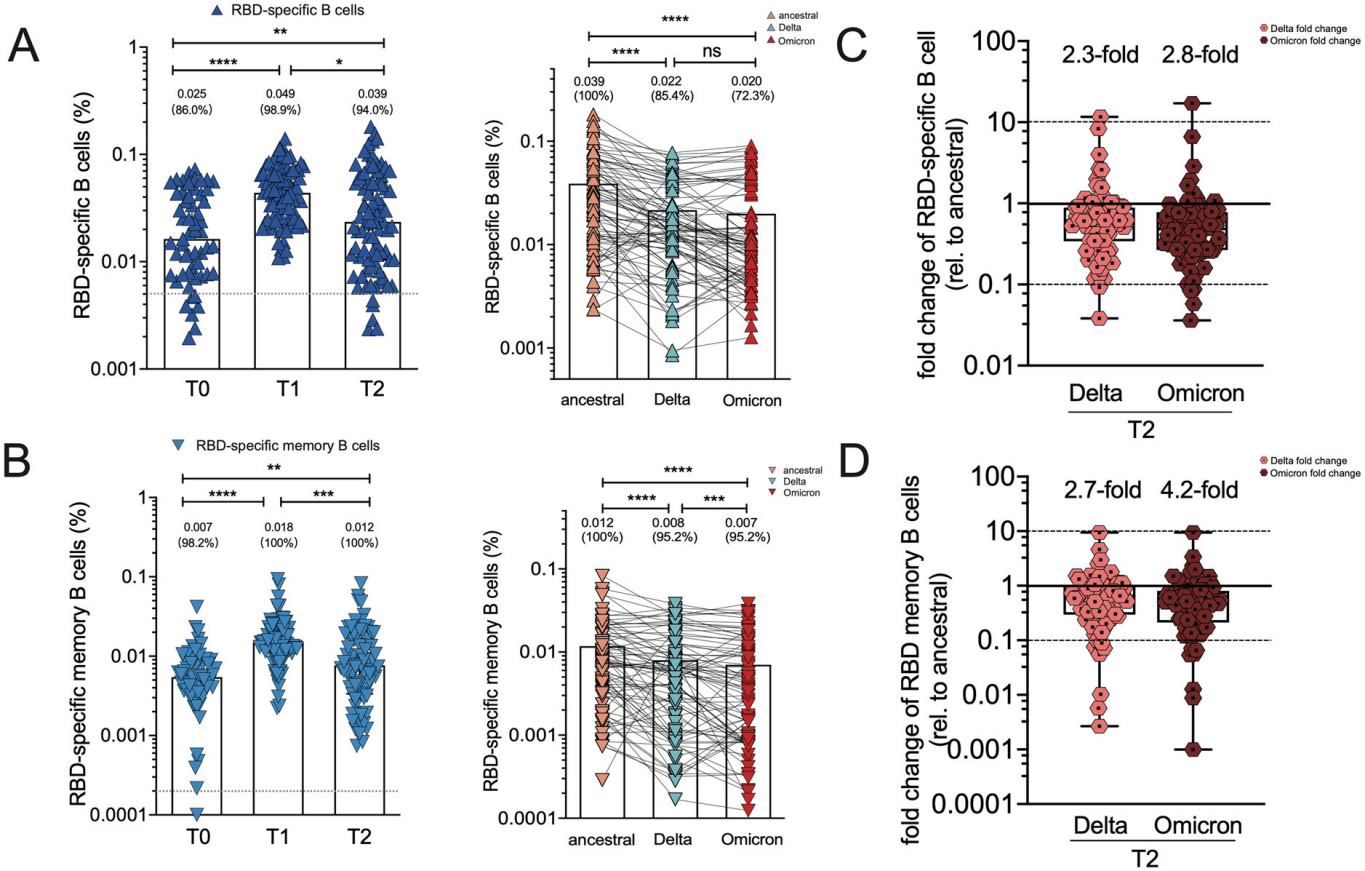
RBD-specific B cell responses and memory B cell subsets in vaccine cohort. (A)The frequency of RBD-specific B cells of total B cells over time in booster recipients at T0, T1 and T2 timepoint, and the frequency of B cells specific to ancestral RBD, Delta RBD, and Omicron RBD at T2 timepoint. Dotted lines indicated the limit of detection (LOD) for the assay. (B) The frequency of RBD-specific memory B cells of total memory B cells over time in booster recipients at T0, T1 and T2 timepoint. The frequency of memory B cells specific to ancestral RBD, Delta RBD, and Omicron RBD at T2 timepoint. (C–D) Fold change for the frequency of RBD-specific B cells (C) and RBD-specific memory B cells (D) recognizing Delta and Omicron strain relative to that of counterpart recognizing ancestral strain. Bars represent the average frequency of B cells, and positive rate was on the top of each bar. Wilcoxon matched-pairs signed-rank with two-tailed *p*-value was used for comparison between groups. * *p* <0.05, ** *p* <0.01, *** *p* <0.001, **** *p* <0.0001; ns, no significant difference.

Two-dose CoronaVac induced potent RBD-specific B cells and memory B cells [21]. Here, the percentages of circulating RBD-specific B cells and its memory B cell subsets in booster recipients were measured**.** At baseline, RBD-specific B cells were still detectable in 86% of individuals. RBD-specific B cells were significantly expanded by 2.65-fold after the booster of CoronaVac, from 0.025% [0.007–0.042] to 0.049% [0.025, 0.067] (*p* < .005), and slightly declined by 2.26-fold to an average frequency of 0.039% [0.010, 0.055] at T2 timepoint (Figure 4(A)). RBD-specific memory B cells (MBCs) represented a large fraction of the neutralizing MBC pool against SARS-CoV-2, expanding substantially by 2.46-fold after the additional dose of CoronaVac (0.007% [0.005–0.009] versus 0.018% [0.015–0.021], *p* < .005), and slightly decreased by 1.7-fold (0.012% [0.009–0.015]) 2-month after the booster dose (Figure 4(B)). Additionally, the recognition breadths of RBD-specific B cells and their memory subsets at T2 timepoint were also analyzed. The 3-dose CoronaVac recipients had 0.022% (0.017, 0.026%) and 0.020% (0.0015, 0.025%) of circulating B cells recognizing Delta and Omicron RBD, respectively, corresponding to 2.3-fold and 2.8-fold reduction in the percentage of VoC RBD-specific B cells compared to that of ancestral RBD-specific B cells (Figure 4(C)). A third dose also resulted in detectable RBD-specific memory B cells recognizing Delta (0.008% [0.006, 0.010%], by a factor of 2.7) and Omicron variant (0.007% [0.005, 0.009%], by a factor of 4.2), respectively, compared to ancestral RBD-specific B cells (Figure 4(D)). Therefore, RBD-specific B cell recognition was also partially affected by emerging VoC strains.

### SARS-CoV-2 spike specific circulating T follicular helper cell responses

**Figure 5.**
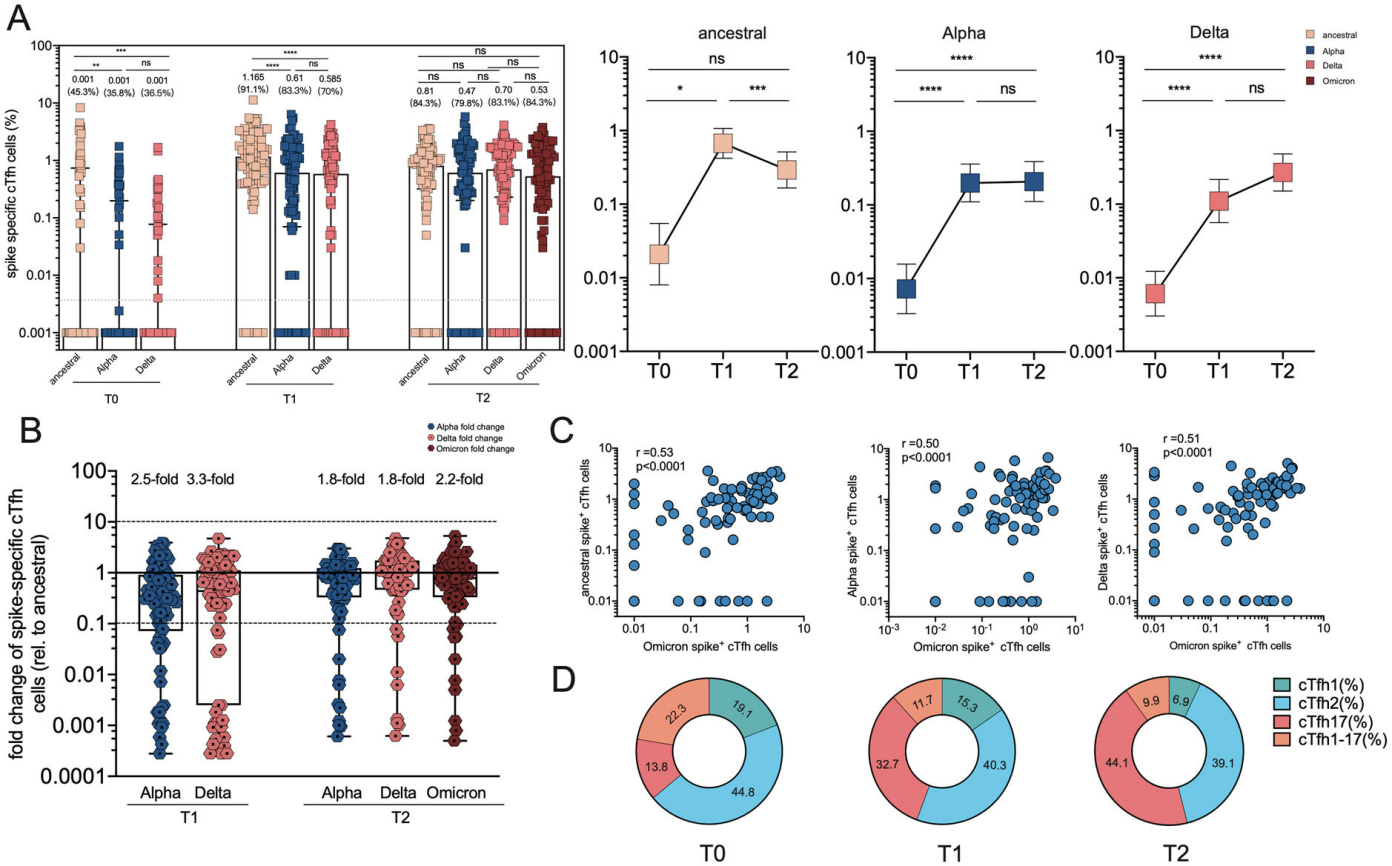
Spike-specific circulating follicular helper cell (cTfh) responses in vaccine cohort. (A) The frequency of cTfh responding to ancestral spike, Alpha spike, Delta spike and Omicron spike (T2 only) of total cTfh cells over time in booster recipients. Dotted lines indicate the lower limit of detection (LOD) for the assay. (B) Fold change for the frequency of cTfh cells recognizing Alpha, Delta and Omicron strain relative to that of counterpart recognizing ancestral strain. (C) Correlation analyses of Omicron spike-specific cTfh cells and ancestral spike-specific cTfh cells, Alpha Spike-specific cTfh cells, and Omicron spike-specific cTfh cells, respectively. (D) Dynamic change of spike-specific cTfh subpopulations at T0, T1 and T2 timepoint, including cTfh1, cTfh2, cTfh17 and cTfh1-17. Bars represent the average frequency of cTfh cells, and positive rate was on the top of each bar. Two-tailed *p* values were determined using matched-pairs signed-rank test with the Holm-Šídák multiple comparison correction between groups. * *p* < .05, ** *p* < .01, *** *p* < .001, **** *p* < .0001; ns, no significant difference.

Tfh cell responses are necessary to form and sustain germinal center (GC) reactions, which are critical to develop long-lasting, high-affinity antibody responses [29,30]. Besides, circulating Tfh (cTfh) cells in the peripheral blood resemble GC Tfh cells and serve as a counterpart to GC Tfh cells to support antibody secretion due to a similar phenotype [31]. Here we sought to track and depict spike-specific cTfh responses over time. Compared to the minimal level of detectable spike-specific cTfh cells at baseline (0.001%), they peaked in peripheral blood 14 days at 1.165% of frequency after CoronaVac boost and remained at a median frequency of 0.81% after 2 months of the third dose immunization (Figure 5(A)). Additionally, they were compared with cTfh responses specific to VoC spike by testing spike peptides corresponding to the viral sequences of the Alpha and Delta strains. Interestingly, a small fraction of responders exhibited a loss of cTfh cell recognition of Delta (7/90; 7.8%) or Omicron (12/90; 13.3%) at T1 timepoint. Slight reductions of cTfh responses to Alpha spike (16%) and Delta spike (9%) were observed. At T2 timepoint, cTfh cell responses specific to Alpha, Delta and Omicron spike remained stable at the frequencies of 0.47%, 0.70% and 0.53%, respectively. The frequencies of Alpha or Delta spike-specific cTfh cells were significantly lower than those of ancestral spike at T1 timepoint (2.5-fold and 3.3-fold, respectively) (Figure 5(A,B)), but not T2 timepoint. The frequency of Omicron spike-specific cTfh cells was highly correlated with the frequency of ancestral spike-specific cTfh cells (*r* = 0.53, *p* < .0001), Alpha spike-specific cTfh cells (*r* = 0.50, *p* < .0001) and Delta spike-specific cTfh cells (*r* = 0.51, *p* < .0001) (Figure 5(C)).

With the purpose of extending these findings, the phenotypic characteristics of SARS-CoV-2 spike-specific cTfh cells were investigated using CXCR3 and CCR6 chemokine receptor markers (Figure 5(D)). CXCR3 and CCR6 were adopted to identify the distinct B cell helper functions, including cTfh1 (CXCR3 ^+ ^CCR6^-^), cTfh2 (CXCR3^-^CCR6^-^), cTfh1-17 (CXCR3 ^+ ^CCR6^-^), and cTfh17 (CXCR3^-^CCR6^+^) subsets [31,32]. cTfh2 and cTfh17 cells can induce B cell differentiation and antibody secretion and regulate immunoglobulin (Ig) isotype switching; cTfh1 cells are commonly considered not to be an effective helper for B cells. At T0 baseline, the phenotypic analysis of total cTfh cells from booster recipients revealed that cTfh1, cTfh2, cTfh17, and cTfh1-17 subsets occupied 19.1% (12.81% to 25.1%), 44.8% (37.2–53.0%), 13.8% (8.0–17.1%), and 22.3% (9.3–31.6%), respectively. Interestingly, boosters exhibited the skewed distribution of cTfh cells toward the cTfh17 phenotype after 2 weeks (32.7%, 28.1–37.5%) and after 8 weeks (44.1%, 39.2–48.6%), whereas cTfh2 subsets remained at a similar proportion. Concurrently, cTfh1 subsets gradually declined to 15.3% (10.1–18.0%) at T1 and 6.9% (5.2–8.6%) at T2 timepoint, while cTfh1-17 subsets decreased to 11.7% (8.5–14.1%) at T1 and 9.9% (7.7–11.9%) at T2**.** Thus, the CoronaVac booster can efficiently expand cTfh17 subsets, contributing to the efficient secretion of IgG and IgA [31].

### SARS-CoV-2 specific CD4^+^ and CD8^+^ T cell responses to different SARS-CoV-2 variants in booster recipients

**Figure 6.**
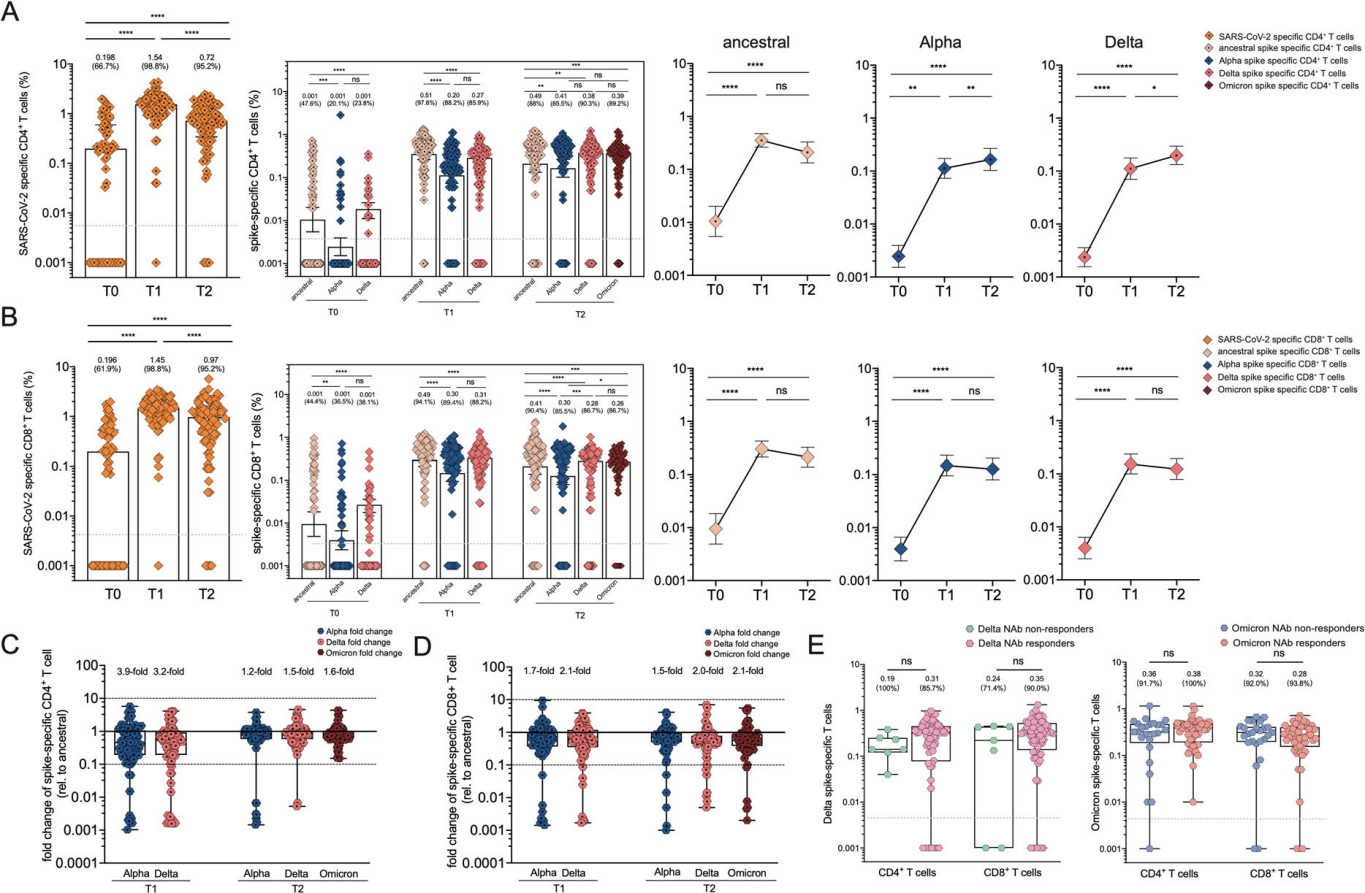
SARS-CoV-2 specific CD4^+^ and CD8^+^ T cell responses in vaccine cohort. (A–B) The frequency of SARS-CoV-2 specific CD4^+^ T cell (A) and CD8^+^(B) T cell responses over time in booster recipients. The frequency of CD4^+^(A) and CD8^+^(B) T cell responses responding to ancestral spike (T0-T2), Alpha spike (T0-T2), Delta spike(T0-T2), and Omicron spike (T2 only) over time in booster recipients. Dotted lines indicated the limit of detection (LOD) for the assay. **(**C-D) Fold change for the frequency of CD4^+^ T cells (C) or CD8^+^ T cells (D) recognizing Alpha, Delta and Omicron strain relative to that of counterpart recognizing ancestral strain. (E) Comparative analysis for the frequency of CD4^+^ T cells or CD8^+^ T cells specific to Delta strain among those booster recipients who do not generate neutralization antibody responses against Delta strain (Delta NAb non-responders) versus Delta NAb responders (left panel). Comparative analysis for the frequency of CD4 ^+ ^T cells or CD8^+^ T cells specific to Omicron strain among those booster recipients who do not generate neutralization activities against Omicron strain (Omicron NAb non-responders) versus Omicron NAb responders (right panel). Bars represented median value, whereas median value and positive rate were on the top of each bar. When comparing T cell responses specific to different SARS-CoV-2 variants and at timepoints, two-tailed *p* values were determined using matched-pairs signed-rank test with the Holm-Šídák multiple comparison correction. Unpaired Wilcoxon test were used for comparison between vaccine booster at T1 timepoint and breakthrough infection subjects. * *p* < .05, ** *p* < .01, *** *p* < .001, **** *p* < .0001; ns, no significant difference.

Beyond antibodies and memory B cells, T cells can contribute to protection upon re-exposure to the virus. Activation-induced marker (AIM) assay was used to measure SARS-CoV-2 CD4^+^ and CD8^+^ T cell responses with the overlapping peptide pools from the ancestral strain. Firstly, SARS-CoV-2 specific CD4^+^ T cells were detected in 66.7% of individuals with an average frequency of 0.198% 9 months after two vaccine doses (Figure 6(A)). A significant elevation to 1.54% for SARS-CoV-2-specific CD4^+^ T cell responses was observed, and such positive responses were detected in 98.8% of individuals after the booster dose. SARS-CoV-2 specific CD4^+^ T cell responses slightly decreased to 0.72% (*p* < .0001) and were detectable in 95.2% of participants at T2 timepoint. Similarly, spike-specific CD4^+^ T cell responses followed a similar trend, except that they remained at a high level 2 months after the booster dose**.** Since T cell responses were less affected by VoCs than humoral immune responses [33,34], the cross-reactivity of CD4^+^ T cell responses to spike proteins derived from different SARS-CoV-2 variants in our cohort was tested. CD4^+^ T cell responses to Alpha and Delta spike were reduced compared to that in ancestral spike at T1 timepoint, as demonstrated by 3.9-fold and 3.2-fold reduction (Figure 6(C)). Meanwhile, a smaller effect of Alpha, Delta and Omicron mutations on CD4^+^ T cell responses was observed at T2 timepoint, compared to that of T1 timepoint, as revealed by 1.2-fold to 1.6-fold change**.** Similar results were observed for CD8^+^ T cell responses at similar frequencies**.** At baseline, SARS-CoV-2 specific to CD8^+^ T cells was detected in 61.9% of individuals with a frequency of 0.196% (Figure 6(B)). It significantly elevated to 1.45% in 98.8% of individuals 2 weeks after booster at T1 timepoint and gradually declined to 0.97% while remaining positive in 95.2% of subjects at T2 timepoint. The magnitude of the SARS-CoV-2 specific CD8^+^ T cell responses against ancestral strain, Alpha variant and Delta variant were significantly boosted from the baseline of 0.001% to 0.49%, 0.30% and 0.31% after the third dose, respectively, and maintained at a similar magnitude at T2 timepoint. The recognition of CD8^+^ T cells to Alpha and Delta spike was decreased by 1.7-fold and 2.1-fold, respectively, at T1 timepoint, and the recognition of CD8^+^ T cells to Alpha, Delta and Omicron spike was slightly decreased by 1.5-fold, 2.0-fold and 2.1-fold, respectively, at T2 timepoint (Figure 6(D)). Additionally, both Delta spike-specific CD4^+^ and CD8^+^ T cell responses were compared between neutralizing antibody (NAb) responders and NAb non-responders in the booster cohort. Regardless of NAb responses against Delta or Omicron strain, there are comparable levels of CD4^+^ and CD8^+^ T cell responses specific to Delta spike or Omicron spike (Figure 6(E)). These results revealed that CD4^+^ and CD8^+^ T cell recognition of VoC spike is largely preserved compared to the ancestral strain.

## Discussions

Substantial efforts have been made to speed up booster vaccination campaigns, given the rapid spread of omicron worldwide. Our understanding of the vaccine-elicited immunological features associated with the main VoCs is the key to informing health policies, including boosting vaccination schedules. This also contributed to the development of potential variant-specific or pan-coronavirus vaccines.

The dynamic humoral and cellular responses in a cohort of CoronaVac booster to emerging SARS-CoV-2 variants, including ancestral, Delta and Omicron strains were analyzed in this study. Consistent with previous reports [34,35], a substantial improved humoral immunity after CoronaVac boost or breakthrough infection was observed in our study. The third dose of CoronaVac significantly increased not only IgG responses but also IgA responses specific to spike protein. Notably, secretory IgA might play an essential role in protecting the mucosal surface against SARS-CoV-2. The booster of CoronaVac enhanced both the seroconversion rate of Delta and Omicron neutralization and the neutralizing potency, highlighting the necessity of a third dose of CoronaVac. In line with a previous report [36], breakthrough infection after two-dose vaccination of COVID-19 inactivated virus vaccine resulted in a natural booster to humoral immunity against SARS-CoV-2. Moreover, breakthrough infection-induced significantly higher neutralization titre against SARS-CoV-2 variants compared to the boosting of CoronaVac, though there are comparable levels of binding antibody titre specific to these VoC antigens. This might be caused by distinct routes of antigen exposure between vaccination and nature infection.

There are still knowledge gaps in our understanding of VoCs regarding the vaccine-elicited cellular immune activity. The role of cellular immune responses and activated T-B cells stimulated by the antigen might be more imperative than circulating antibodies. Circulating spike-specific B cells may have crucial contributions to protective immunity by making anamnestic neutralizing antibody responses after infection. The continued maturation of B cell responses over time would assist in adapting SARS-CoV-2 immunity to VoCs [37]. Reduced B cell binding of RBD protein from variants was observed in all cases, while the reduction was less than 5-fold for Delta spike and Omicron RBD protein. This demonstrated a partial retained B cell recognition of variants, consistent with the observations that Omicron neutralizing antibody titres rapidly increased after the third immunization or breakthrough infection but were generally low among individuals with two-dose of CoronaVac.

The direct evaluation of key Tfh immunological events in lymphoid tissues after immunization is challenging in humans, making surrogate biomarkers such as cTfh cells in the blood especially informative [39]. In COVID-19 recovered individuals, spike-specific cTfh differentiated subjects were associated with potent neutralizing responses [40]. Robust Tfh cells were detected in paired blood and lymph node specimens from SARS-CoV-2 mRNA vaccinated individuals [41], which persisted at a nearly constant frequency for at least six months. Similarly, our study revealed that the third dose of CoronaVac induced robust and persistent spike-specific cTfh cell responses, which were correlated with the vaccine-induced RBD-specific B cells and serum neutralization potency. We firstly verified that the expanded cTfh cell responses induced by CoronaVac booster exhibited a clear phonotypic bias toward a pro-inflammatory Tfh17 subset, previously reported for other viral glycoproteins [42]. Additionally, the magnitude of spike-specific cTfh cells remained unchanged and was less sensitive to mutations within VoC spikes, ranging from a 1.8-fold to 3.3-fold decrease. Therefore, the booster dose-induced cTfh cells can rapidly expand and further facilitate memory B cells to evolve, providing effective humoral responses upon virus re-exposure.

Distinct from B cell and cTfh cell recognition, our data suggested that the third dose of CoronaVac elicited broadly cross-reactive cellular immunity against SARS-CoV-2 variants, including Delta and Omicron. The magnitude of Omicron cross-reactive T cells was comparable to that of Alpha and Delta variants, though the Omicron spike has a greater number of mutations. Our observation was also in good agreement with previous studies that the effect of variant mutations on global CD4^+^ and CD8^+^ T cell responses was negligible [43–45] due to highly conserved CD4^+^ and CD8^+^ T cells epitopes within the viral variants [46]. The mutations derived from Delta and Omicron extended a limited impact on T cell responses, suggesting that vaccination or prior infection could provide substantial protection from severe disease. Indeed, these well-preserved T cell immunity to Delta or Omicron acquired through vaccination or infection might contribute to protection from severe COVID-19, consistent with lower risk of hospitalization and reduced disease severity observed in recent Omicron wave from South Africa [47].

Currently, the correlation of protection for a vaccine against SARS-CoV-2 remains elusive, though the humoral and cellular responses induced by vaccines are well characterized. Neutralizing antibodies could serve as a correlate of protection for vaccines against SARS-CoV-2, while antibody testing might lead to misperception and misunderstanding of vaccine effectiveness among the general population. Our study revealed that those NAb non-responders also had a similar magnitude of T cell responses, compared to NAb responders, suggesting that those without neutralizing antibody responses also benefited from the vaccine owing to robust and persistent T cell responses acquired by immunization.

This study has several limitations. First, this study was deficient in the data on the long-term follow-up of humoral and cellular responses after the third boost of CoronaVac. Follow-up studies should be conducted to monitor the duration and persistence of adaptive immune response. Additionally, we did not characterize the phenotypic memory differentiation for SARS-CoV-2 specific CD4^+^ and CD8^+^ T cell responses, nor test SARS-CoV-2-specific T cells responses in the breakthrough infection cohort. Thus, longitudinal T cells responses elicited by either booster vaccination or breakthrough infection should be compared in future studies.

To summarize, our study highlighted that a booster dose of CoronaVac can provide give a significantly larger boost for the neutralizing antibody responses and cellular responses that cross-recognize Delta and Omicron variants, compared to the two doses of vaccine. Moreover, the potency, breadth, and duration of adaptive responses improved concomitantly. Nevertheless, the data also underlined the need for continued surveillance and the potential danger posed by continued variant evolution that resulted in further reduction of adaptive immune responses. The incorporation of additional elements eliciting broader adaptive immune responses directed towards more conserved targets into vaccine strategies may be considered a means to increase vaccine effectiveness against future variants.

## Supplementary Material

Supplemental Material
