# Supplementary material for: The Third dose of CoronVac vaccination induces broad and potent adaptive immune responses that recognize SARS-CoV-2 Delta and Omicron variants
Source: Emerg Microbes Infect. 2022 Jun 2;11(1):1524–36. doi: 10.1080/22221751.2022.2081614 (PMC9176682; doi:10.1080/22221751.2022.2081614)
Supplement: Supplemental Material [file TEMI_A_2081614_SM1740.docx]

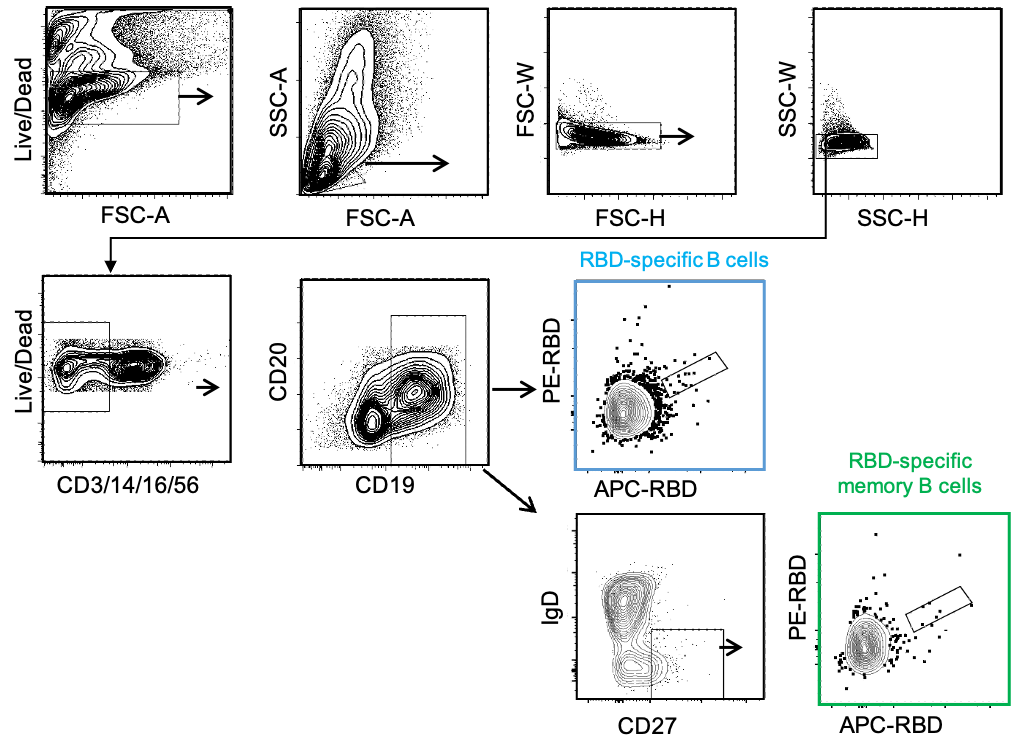


**Supplemental Figure 1. Gating strategies to define SARS-CoV-2 RBD-specific B cells.** Live cells were identified as Live/Dead^-^ and lymphocytes were then gated based on forward- and side-scatter. Doublets were then excluded by FSC-W vs. FSC-H and SSC-W vs. SSC-H. Total B cells were identified as CD3^-^CD14^-^CD16^-^CD56^-^CD19^+^CD20^+^cells. Memory B cells were identified as CD20^+^CD27^+^cells. RBD specific B cells were identified based on binding to corresponding RBD probes.


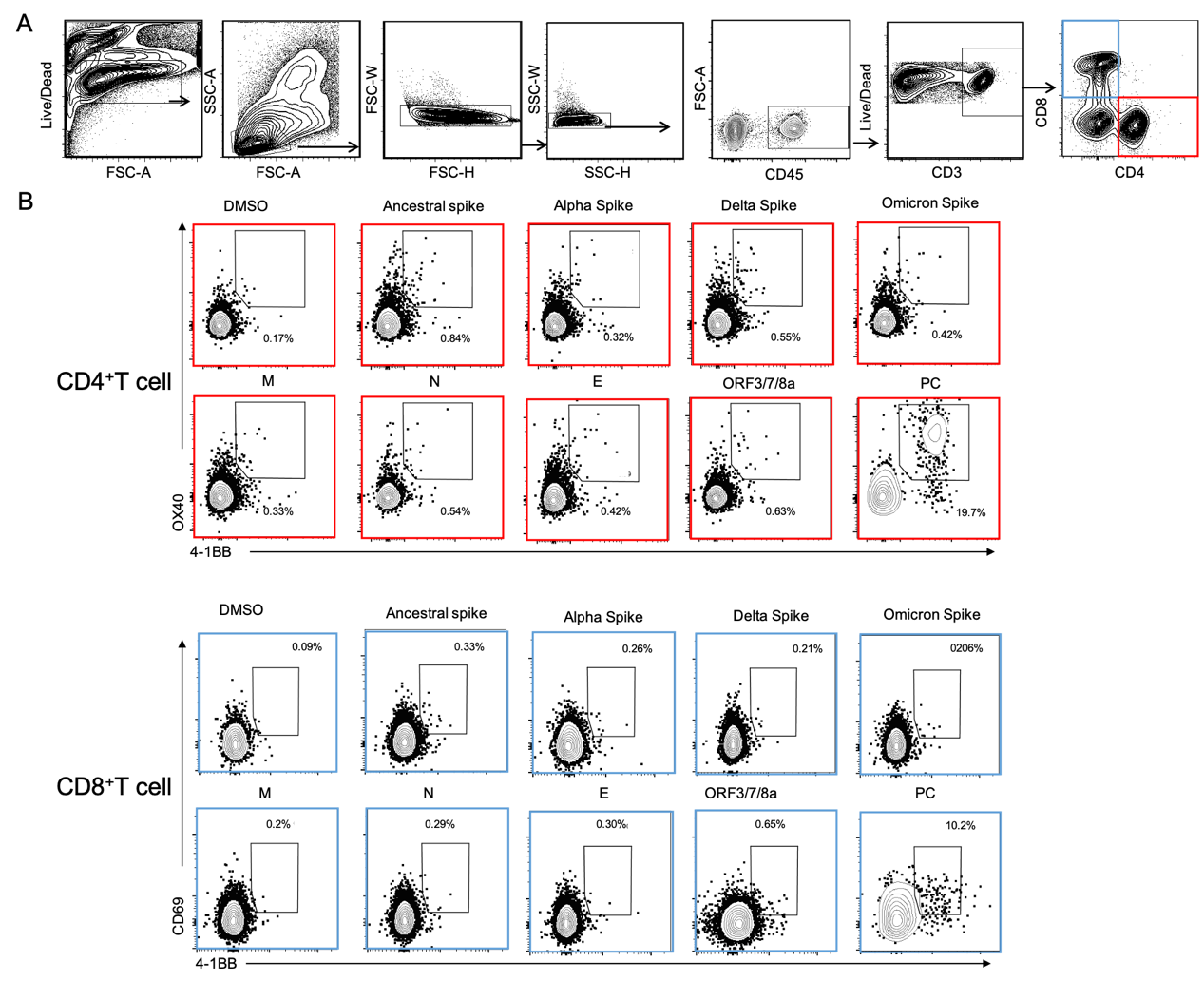


**Supplemental Figure 2. Gating strategies to define SARS-CoV-2 specific T cells. (A)** Live cells were identified as Live/Dead^-^ and lymphocytes were gated based on forward- and side-scatter. Doublets were then excluded by FSC-W vs. FSC-H and SSC-W vs. SSC-H. Total T cells were identified as CD45^+^CD3^+,^ which were further divided into CD8^+^ and CD4^+^ subsets. **(B)** Representative examples of flow cytometry plots of SARS-CoV-2-specific CD4^+^ and CD8^+^ T cells by activation-induced marker (AIM) assay. AIM^+^CD4^+^ T cells were identified based on dual expression of 4-1BB and OX40, while AIM^+^CD8^+^ T cells were identified based on dual expression of 4-1BB and CD69, after overnight stimulation with indicated peptide pools including ancestral spike, Alpha Spike, Delta Spike, Omicron Spike, Membrane (M), Nucleocapsid (N), membrane protein (M), ORF3/7/8a peptide pools or PMA/Ionomycin as positive control (PC), compared to negative control stimulation (DMSO).

**
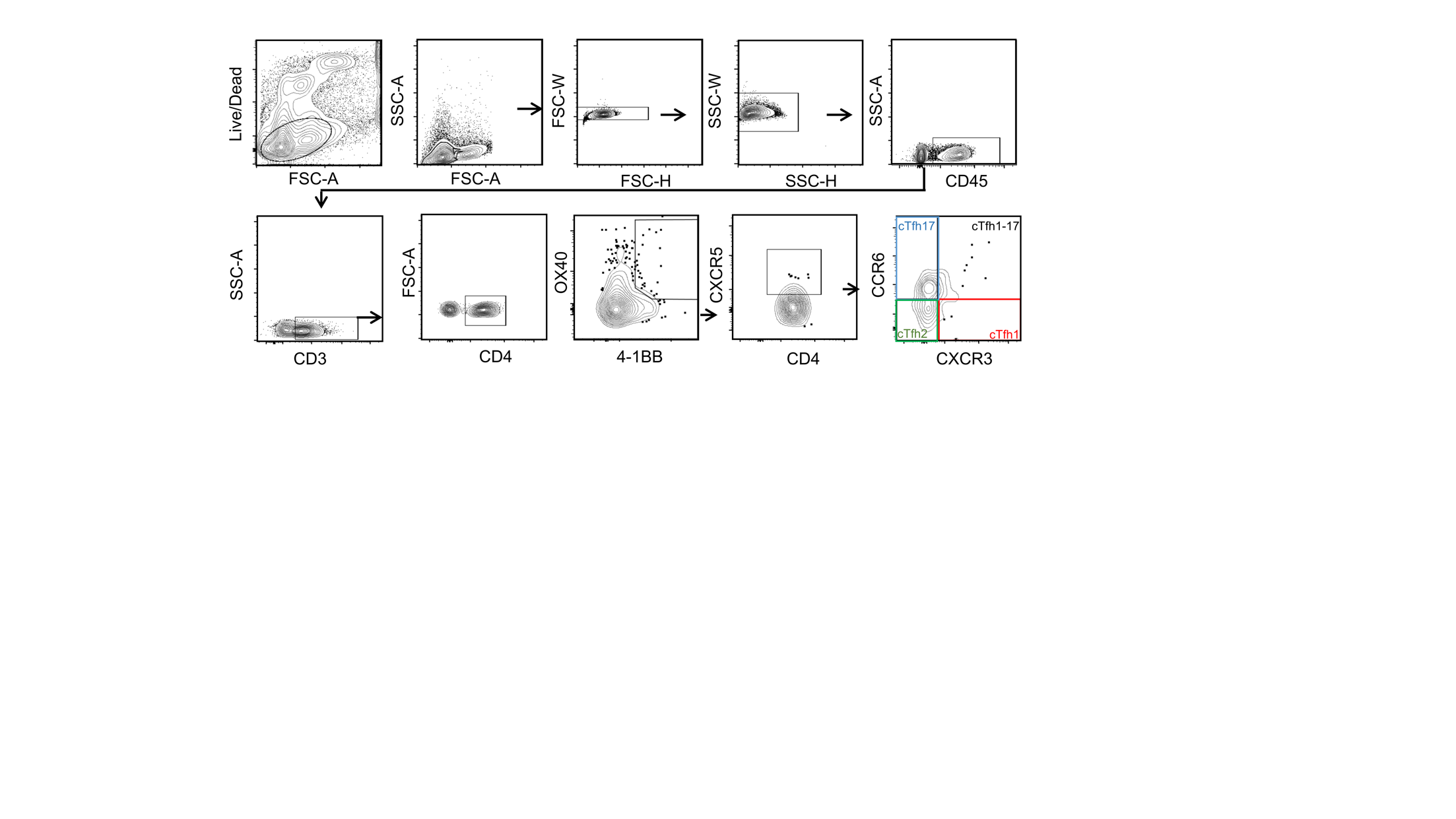
**

**Supplemental Figure 3. Gating strategies to define SARS-CoV-2 spike specific circulating Tfh (cTfh) cells.** Live cells were identified as Live/Dead^-^ and lymphocytes were gated based on forward- and side-scatter. Doublets were then excluded by FSC-W vs. FSC-H and SSC-W vs. SSC-H. Total T cells were identified as CD45^+^CD3^+^. After overnight stimulation with the indicated spike peptide pools, CD4^+^ T cells with the dual expression of 4-1BB and OX40 were considered as AIM^+^CD4^+^ T cells. Spike-specific cTfh cells were gated as CXCR5^+^4-1BB^+^OX40^+^CD4^+^ cells and further divided into Tfh1(CXCR3^+^CCR6^-^), Tfh2(CXCR3^-^CCR6^-^), Tfh17(CXCR3^-^CCR6^+^) and Tfh1-17(CXCR3^+^CCR6^+^).
